# The Nature and Development of Accidental Productions

**Published:** 1845-10

**Authors:** Ch. Baron


*The Nature and Development of Accidental Productions.* By Ch. Baron, M.D.—This communication is part of an entire work upon the subject to which it relates, presented some time since to the Academy. That portion of it which relates to *Tubercle* has already appeared in the Archives Generales de Médecine, for 1839.

Before considering any of their varieties in detail, the author first states his reasons for considering *all Accidental Productions are of an identical nature and origin.*

1. *The frequent similitude of anormal tissues.*—This is sometimes so great, in some of the intermediate stages of morbid growth, that one is with great difficulty distinguished from another. Thus we may be at a loss to decide whether certain visceral changes should be referred to unsoftened encephaloid, to scirrhous, or to fibrous tissue. The same may often be observed in respect to tubercle and encephaloid in a state of crudity. In some organs the difficulty is greater than in others, and, as M. Cruveilhier observes, nothing is more difficult than the classification of tumours of the brain.

2. *The mutual Transformations which are sometimes operated among some of these productians.*—Thus pus may be converted into a false membrane, cancer into pus, hydatids into tubercle or cancer, cartilage into bone, and tubercle into stony concretion.

3. *Their simultaneous Appearance.*—The author does not agree in the opinion still held by some observers, that different morbid tissues exclude each other. Different morbid products may be observed in different organs, in the same organ, or even in the same tumour. One accidental production may even be developed within the interior of another: *e. g.* tubercles within the substance of false membranes.

4. " *Their Analogy of Structure.*—The masses in all these productions are constituted of small tumours, which we may call initial, and which, in most instances, approach the spherical form. They are formed of various superimposed layers, which seem to converge towards the centre. Frequently the colour and density are not alike throughout the extent of the tumour, the centre being usually the least consistent part. Around accidental tumours is a vascular network, whence branches are sent into their substance in which many are lost, and frequently, in following the vascular ramuscules which penetrate the morbid tumour, we observe they terminate in one of the little granules, the assemblage of which constitutes the mass."

5. *Their Organization and Vitality.*—The majority of these productions are organized, and thus may undergo changes in volume, suppuration, &c. Their vitality, which is restricted, and not alike in all, is due to the vessels which supply them with the material of their nutrition, and maintain their relation to the general circulation.

6. *The Analogy of their Development.*—Most productions are first visible as white or yellowish-white points. Their consistence increases faster than their volume or colouration. This is their period of crudity, which is wanting in some, as pus. Sooner in some than n others the period of softening occurs, commencing in the centre.

A more minute inspection shows that, in the earliest stage of their existence, the greater part of these productions are in the fluid state.

7. *The Analogy of Seat.*—Organs well supplied with vessels are those which are liable to accidental productions, and it is in their most vascular portions that these are developed. They are not found, however, in the vicinity of the large, but in that of the small vessels; *e. g.* it is not in the vicinity of the roots of the lungs that the morbid productions of these organs are deposited, while it is the corticle substance of the kidney that is affected with granulations.

8. *Their Generalization.*—The disposition to this is so great, that one may almost say it is more common to find several simultaneous depositions of a given accidental production than to find one only.

9. *The Condition of the surrounding Tissue* is nearly alike in all. During the period of crudity the fibres of this undergo only slight separation and compression, but during the period of softening it offers the characters of chronic inflammation. The morbid product, at the period of its origin, is surrounded by an areolar injection, which has been erroneously considered inflammatory. Sometimes the neighbouring vessels are found to contain more or less coagulated blood.

10. *The Analogy of Symptoms.*—The local symptoms, depending on the obstruction the deposit causes in the organ, are very similar in the various accidental productions, and these cannot be distinguished form each other by their means. The general symptoms, especially manifesting themselves during the period of softening, although they occur at different stages of the disease in different cases, bear a great resemblance to each other, and are especially characterized by the exhaustion of the powers of the frame.

11. *The Ætiology is frequently the same*, as shown in the effects of hereditary influence, inflammation, congestion, contusions, &c.

12. *The Chemical Composition* is remarkably similar—the differences being almost wholly due to the varying proportion of the same elements.

"The reasons which I have now assigned seem to me to sufficiently prove the identity of the origin and nature of accidental productions. What I wish now to show is *the identity of non-analogous morbid productions and of analogous morbid productions.* The propositions above enounced nearly equally apply to these two classes of productions, and we may refer especially to the frequent simultaneous existence of analogous and non-analogous degenerescences. Thus, one often meets with, in the same subject, and often in the same tumour, encephaloid matter, tubercular and osseous, scirrhous and fibrous tissue. Considering, then, these analogous and non-analogous productions identical, it results that the origin of morbid tissue is the same as that of the normal tissues. It is, indeed, impossible to deny that accidental cellular and tendinous tissue is identical with normal cellular and tendinous tissue, and as we admit the analogy of the analogous and non-analogous morbid products, we must admit the community of origin of normal and anormal tissue. Professor Bouillaud observes, 'the laws which preside over the formation and development of the anormal tissues are but modifications of those to which the formation and developement of normal tissues are submitted.' This analogy is further proved by the vascularity and evident organization of the accidental productions, their participation in the general modifications of the organism, and their vitality."

Trousseau and Leblanc, and others, have referred the origin of these productions to a *sub-inflammation ;* but no inflammatory process is usually set up around them unless and until softening occurs, and, if it existed, would produce very different results, accordingly as these productions were situated in one organ or another. The opinion of Cruveilhier, that they are due to a *phlebitis*, because coagula are sometimes found in adjoining vessels, is not more tenable.

These anormal tissues are generated from the *blood*, and therefore an afflux of this fluid, or even in some cases inflammatory action, is favourable to their production. It is difficult to go beyond mere hypothesis in stating how these changes in the fluid are affected. However, the developement of certain products from effused and coagulated blood has been observed. "We see the blood arrested in its vessels or effused into the tissues, and the resulting coagula themselves induce a true secretion, a separation of the elements necessary for the formation of the morbid production. Certain elements of the blood disappear by absorption, and others are subjected to successive transformations, whence result the anormal products." The names of Abernethy, Forget, Andral, and Piorry, are cited in proof of this generation of anormal productions in coagula.

*Encephaloid Matter.*—After describing several autopsies, in which the gradual conversion of a coagulum into encephaloid matter was observed, the author thus sums up his observations:

"In a great number of cases a coagulum is deposited in the organic tissue or is yet contained in the vessels. The serum is first absorbed, and the colouring matter is removed, the concretion becoming fibrinous, yellowish, then whitish, and augmenting more and more in consistency. The matter then becomes smooth and lardaceous like dense cerebral matter, a slight rose colour pervading it. The degeneration is usually more advanced as we approach its centre, so that sometimes the encephaloid matter is completely formed and even softened at the centre, while the circumference is yet constituted of fibrinous blood, or of blackish red blood, the consistency of gooseberry jelly. At a later period, the external layer of the production may become converted into an enveloping cyst, or may act as a partition in a mass when many tumours are conjoined. The cyst is often, however, formed of the cellular tissue of the organ in which the tumour is deposited.

"The mass increases by the addition of new material which the numerous vessels that traverse it supply, by the conversion into encephaloid of the effusions of blood which occurs in its various portions, especially towards its centre, and after by the union of many neighbouring tumours."

After offering a similar explanation of the origin of *colloid* or *gelatinous cancer*, the author next presents several observations upon

*Hydatids of the Lungs and Pulmonary Apoplexy.*—The blood effused into the texture of the lung, constituting pulmonary apoplexy, when not absorbed, may undergo great change. The most excentric layer of the effusion may have undergone absorption, leaving a yellowish intermediate layer containing blood in its cavity. Sometimes, as this fluid internal portion of the blood disappears, this yellow envelope contracts and remains a persistent cicatrix. In other cases the fluid is removed from the centre by communications with the bronchi, or by the interior of the envelope taking on the secretory action, and a hydatid cyst is left, which becomes lined with a smooth polished membrane, and filled with a serous fluid.

"I add, as a proof in support of this mode of the origin of hydatids, that a common cause of their development is a fall or shock, such as may produce a commotion and effusion of blood in the organ which contains them. Further, we often see morbid tumours constituted at once of serous sacs and of other accidental productions, due generally, as I believe I have shown, to the transformation of the effused blood. This simultaneousness of development in the same parts of the economy seems to prove that the acephalocysts and other accidental productions have a common origin. Lastly, as M. Berard states, it is in the spongy portion of the long, and the diploe of the flat, bones, *i. e.* in the most vascular portions, that hydatids ordinarily develop themselves. So, too, within the cranium, it is chiefly near the pia-mater, the vascular network enveloping the brain, that hydatids arise, and especially in the fissures, in which are lodged the principal arterial branches.

"To those who may feel surprised that the most excentric layers of a coagulum should be connected with an hydatid cyst, non-adherent to the cavity containing it, I may answer that the walls of the acephalocyst possess all the properties of fibrine and albumen, and that, since the work of Legroux on Sanguine Concretions, it is impossible to doubt that a coagulum may be converted into a cyst; and that M. Bouillaud, among many other authors, cites numerous enough examples of coagula being so converted into simple or compound cysts. We must not regard the transformation of the pulmonary apoplectic deposits as a fiction, drawn from a rational induction, but as a fact resulting from observation; for, as cerebral apoplectic *foyers* are frequently converted into serous cysts, the occurrence of the same thing in the lung can hardly be disputed."

*Melanosis.*—After citing the observations of several authors, to prove that melanosis is produced by a transformation of the coagulum of the blood, the author observes:

"The chemical composition of melanosis has thrown much light on its nature. All chemists who have analyzed this matter have remarked its great analogy to the blood. It is seen by the analysis I have given above, that fibrine, albumen, oxide of iron, and many salts, are found in it in notable proportions, and very similar to those which exist in the blood. But there is a principle to which we must direct some attention, namely *carbon*, which is found so abundantly in melanosis as to constitute one-third of its substance. As this principle only exists in small proportion in the blood, a supplementary quantity must be formed to constitute melanosis. The proportion of carbon is probably not the same in all melanoses, being feebler in those which are soft and reddish brown, than in those which are black, dry, and dense. It is easy to prove that melanosis is constituted by the combination of most of the elements of the blood with a considerable quantity of carbon, but it is not so to discover the origin and mode of formation of this carbon. According to MM. Barruel, Foy, Trousseau and Leblanc, the cruor is the only element of the blood whose change can furnish it. This theory of its origin is a rational one. Nevertheless, I believe carbon may also be derived from other sources. Thus, in the lungs, a portion may be derived from the chemical act of respiration. A part retained in the lungs mixes with the blood, whose coagulation it produces, and from its mixture with this coagulated blood, results the melanosis. The carbon of pulmonary melanosis may also be in part produced by the combustion of bodies employed in artificial illumination."

*On the Black Pulmonary Matter.*—The author, in concluding his paper, details the usual appearances which the deposition of the black pulmonary matter presents. It is found at the surface of the lungs, either in the form of mere points, lines, or superficial layers. On examining the interior of the organ, points and lines of black substance are found chiefly in the interlobular tissue, and augmenting in size as they approach the roots of the lungs. Most of these become continuous with small vessels which have exactly the appearance they would have if injected with some solidified black matter. In the bronchial glands at first a black point is observed, then layers of black matter, and at last a sort of general infiltration, which hardly allows the normal tissue to be distinguished. Sometimes it is deposited in amorphous masses.

Dr. Baron considers this black pulmonary matter to be primarily deposited within the bronchial veins; and that its presence in ripe and advanced age is one of the causes to which the diminution of the respiratory powers may be attributed. He regards it as coagulated blood conjoined with carbon. The coagulation may be produced by the presence of carbon, which may be derived from the chemical act which constitutes hæmatosis, and from the combustion necessary for artificial illumination.—*Ibid.*

